# Supplementary material for: Applying GRADE-CERQual to qualitative evidence synthesis findings–paper 6: how to assess relevance of the data
Source: Implement Sci. 2018 Jan 25;13(Suppl 1):4. doi: 10.1186/s13012-017-0693-6 (PMC5791042; doi:10.1186/s13012-017-0693-6)
Supplement: Supplementary file 2 — External relevance of data. (PDF 643 kb) [file 13012_2017_693_MOESM2_ESM.pdf]

## Additional File 2: External relevance of data

For completeness, we briefly discuss external relevance (associated with such concepts as applicability, transferability, generalisability or validity) in order to differentiate how assessments of internal and external relevance are conceptualised within the context of CERQual. These terms are discussed in the context of systematic reviews of complex interventions [1].

The assessment of relevance in CERQual seeks to establish the internal relevance of data contributing to findings in relation to the context(s) specified in the question. However, assessment of the external relevance of evidence is commonly discussed in the methodological literature. This literature describes external relevance or validity as the degree of fit of the structured review question and finding(s) to an external context. The issue of "relevance" is therefore often evoked when seeking to establish the extent to which findings from qualitative research can be generalized or transferred to settings other than those included in the original research [2].

As discussed in this paper, wider external relevance of a review finding is addressed in part by the overall CERQual assessment. This overall assessment seeks to establish the extent to which a synthesis finding is a reasonable representation of the phenomenon of interest [3, 4]. An overall CERQual assessment communicates the extent to which the synthesis finding is likely to be substantially different from the phenomenon of interest, as defined in the review question. Where CERQual is applied to the findings of a published review but with adaptation of the context specified in the original review question to reflect a particular new context of interest (for example, from a publicly funded to a privately funded health system), considerations of external relevance are also important.

While some methodologists argue that qualitative research does not aspire to generalisability [5], others have noted that research that is not considered to be generalizable is of little use, and is unlikely to receive funding [6]. However, quantitative and qualitative approaches differ in the way that they address generalizability. In quantitative work generalizability is statistical, i.e. the study sample is matched to the wider study population to ensure comparability of demographic characteristics. If the sample achieves representativeness and the study is adequately powered with sufficient numbers of participants, then it is assumed that the findings from the sample are generalizable. In some types of qualitative inquiry, however, participants are selected by means of theoretical sampling, i.e. for their ability to contribute information (and consequent theory development) to the area under investigation. Thus Popay and colleagues emphasise that, '…the aim is to make logical generalizations to a theoretical understanding of a similar class of phenomena rather than probabilistic generalizations to a population' [7].

Other studies adopt a more pragmatic atheoretical approach, seeking "representativeness" to facilitate transferability. In short, the intention is to seek commonality of "situation" rather than demographic representativeness. This immediately implies that relevance in a qualitative context could extend beyond the demographic characteristics embodied in a structured review question formulation to embrace wider concerns of situation or context. This, in turn, emphasises the importance of unpacking and making 'context' transparent (with regard to the perspective or population, phenomenon of interest, setting) when specifying the question for a qualitative evidence synthesis, and in the subsequent assessment of the relevance component of CERQual.

Green and Thorogood suggest various ways in which the findings from qualitative research are considered more widely relevant [8]. They refer both to conceptual generalisability and to the more instrumental transferability of findings to similar contexts. Conceptual generalisability is informed,

and may be enhanced by, the use of theory. When findings are supported by a well-developed and evaluated theory, overarching generalities can be identified and explained that transcend time, place and context [9]. A finding may also contribute to further development of the theory.

Conceptual generalisability, at a programme theory level, is illustrated by the development of a logic model in a qualitative evidence synthesis that sought to explain identified barriers and facilitators to implementation of lay health worker programmes [10]. However, CERQual has yet to be applied to syntheses where conceptual or theoretical models were developed from review findings. At present, transferability, with its emphasis on the extent to which the findings of a review can be applied to other contexts similar to those specified in the review question, is central to use of the CERQual approach.

## References


1. Burford B, Lewin S, Welch V, Rehfuess E, Waters E: **Assessing the applicability of findings in systematic reviews of complex interventions can enhance the utility of reviews for decision making**. *Journal of clinical epidemiology* 2013, **66**(11):1251-1261.
2. Finfgeld-Connett D: **Generalizability and transferability of meta-synthesis research findings**. *J Adv Nurs* 2010, **66**(2):246-254.
3. Lewin S, Bohren M, Rashidian A, Glenton C, Munthe-Kaas HM, Carlsen B, Colvin CJ, Tuncalp Ö, Noyes J, Booth A *et al*: **Applying GRADE-CERQual to qualitative evidence synthesis findings - paper 2 of 7: how to make an overall CERQual assessment of confidence and create a Summary of Qualitative Findings table**. *Implementation Science* 2017, **12 Suppl 2**.
4. Lewin S, Glenton C, Munthe-Kaas H, Carlsen B, Colvin CJ, Gulmezoglu M, Noyes J, Booth A, Garside R, Rashidian A: **Using qualitative evidence in decision making for health and social interventions: an approach to assess confidence in findings from qualitative evidence syntheses (GRADE-CERQual)**. *PLoS Med* 2015, **12**(10):e1001895.
5. Horsburgh D: **Evaluation of qualitative research**. *J Clin Nurs* 2003, **12**(2):307-312.
6. Morse JM: **Qualitative generalizability**. *Qualitative health research* 1999, **9**(1):5-6.
7. Popay J, Rogers A, Williams G: **Rationale and standards for the systematic review of qualitative literature in health services research**. *QualHealth Res* 1998, **8**(1049-7323 (Print)):341-351.
8. Green J, Thorogood N: **Qualitative methods for health research**: Sage; 2013.
9. Daly J, Willis K, Small R, Green J, Welch N, Kealy M, Hughes E: **A hierarchy of evidence for assessing qualitative health research**. *Journal of clinical epidemiology* 2007, **60**(1):43-49.
10. Glenton C, Colvin CJ, Carlsen B, Swartz A, Lewin S, Noyes J, Rashidian A: **Barriers and facilitators to the implementation of lay health worker programmes to improve access to maternal and child health: qualitative evidence synthesis**. *The Cochrane database of systematic reviews* 2013, **10**:CD010414.
